# Exploring the adoption of ChatGPT in academic publishing: insights and lessons for scientific writing

**DOI:** 10.3325/cmj.2023.64.205

**Published:** 2023-06

**Authors:** Jan Homolak

**Affiliations:** 1 Department of Pharmacology, University of Zagreb School of Medicine, Zagreb, Croatia; 2 Croatian Institute for Brain Research, University of Zagreb School of Medicine, Zagreb, Croatia

The recent surge in the use of artificial intelligence (AI) and natural language processing technologies holds significant potential for revolutionizing many industries, including scientific writing and academic publishing (1). One notable example is the development of ChatGPT, a widely used large conversational language model with an outstanding capacity to generate human-like text responses. Despite ChatGPT's current limitations in accurately conveying complex scientific concepts and information without substantial human intervention (2), it has considerable potential to enhance academic writing. ChatGPT might help researchers effectively and clearly communicate their findings, thereby improving the allocation of limited resources (1,3). Nevertheless, the integration of ChatGPT in academic publishing remains ambiguous due to ethical dilemmas that have yet to be addressed. For example, an AI model, due to its inability to be held accountable, cannot be acknowledged as an author of scientific articles. Furthermore, the ownership rights and responsibility for AI-generated work, including the possibility of containing plagiarized content, are yet to be determined (1,4). Currently, academic publishers only allow the use of ChatGPT and similar tools to improve the readability and language of research articles. However, the ethical boundaries and acceptable usage of AI in academic writing are still undefined, and neither humans nor AI detection tools can reliably identify text generated by AI (5).

The primary aim of this case study was to examine the utilization of ChatGPT in academic writing and to appraise the dependability of existing AI detectors. Furthermore, the study aimed to foster discussion regarding the ethical limits of employing AI in academic writing and the capacity of publishers to identify transgressions of these limits.

To ascertain the level of AI utilization in composing scientific abstracts, the ZeroGPT AI detection algorithm was employed on a data set containing the most recent 200 abstracts related to Alzheimer disease (AD) sourced from the Scopus database. The ZeroGPT score was converted into binary form to determine the proportion of abstracts belonging to original research and review articles where AI tools had been employed. Surprisingly, 59.5% of original articles and 79.2% of reviews had ZeroGPT scores above 0, a finding indicating prevalent AI usage (Figure 1A). Notably, the extent of AI use was greater in the abstracts of review articles, as evident from higher ZeroGPT scores (Figure 1B). Due to the unexpectedly high scores, two additional AI detection tools, the OpenAI classifier and GPTZero, were employed on the same data set. Interestingly, both classifiers provided more conservative estimates, suggesting a lower number of abstracts that were modified by AI (Figure 1C).

**Figure 1:**
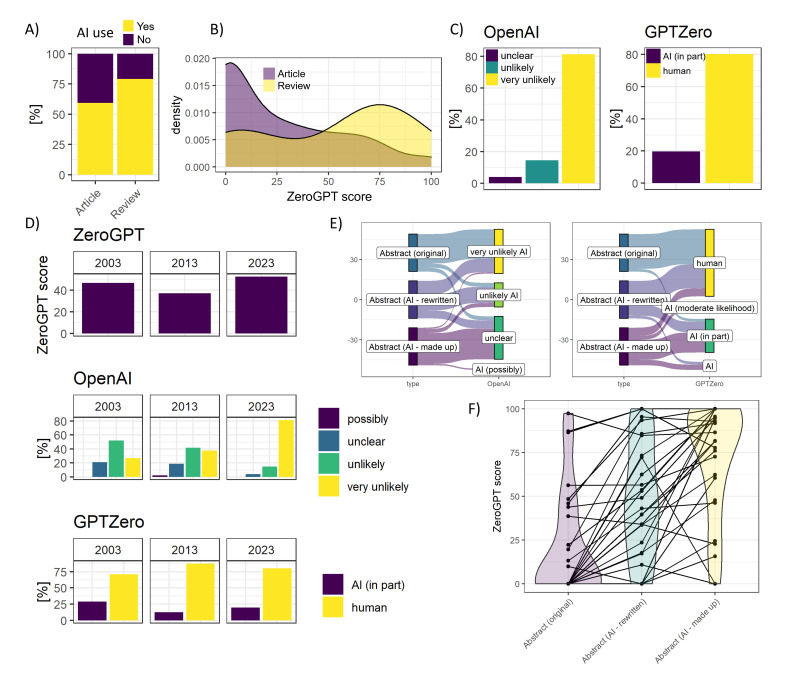
Assessment of the adoption of artificial intelligence (AI) tools in academic writing. (**A**) The utilization of AI tools was examined in the 200 most recent abstracts on Alzheimer disease (AD) obtained from the Scopus database. Inclusion criteria considered the study type (original article, review), while insufficiently lengthy or missing abstracts were excluded. The analysis comprised 148 original articles and 24 reviews, forming the first experiment. (**B**) The distribution of ZeroGPT scores for both the original articles and reviews included in the first experiment. (**C**) The same data set from (**A**) and (**B**) subjected to analysis with the OpenAI classifier and GPTZero. (**D**) Top: The average ZeroGPT scores of AD abstracts from 2003 (n = 48), 2013 (n = 48), and 2023 (n = 172). Middle: The OpenAI classifier scores for articles from the same data set. Bottom: The GPTZero scores for articles from the same data set. (**E**) Sankey plots demonstrating the performance of the OpenAI classifier and GPTZero on a data set consisting of the author's original abstracts (true negatives, n = 28), the same abstracts rewritten by ChatGPT (n = 28), and true positive control abstracts created by ChatGPT based solely on the suggested author and title (n = 28). (**F**) The ZeroGPT scores for the same abstracts analyzed in (**E**).

To verify the reliability of all three detection tools, an additional control experiment was conducted involving two additional smaller data sets containing 100 AD abstracts published in 2003 and 2013, respectively. As in this period, AI tools were not widely available, the procedure allowed for an indirect assessment of the false-positive rate. Surprisingly, all three algorithms indicated that AI use in scientific abstract writing remained relatively stable over the past two decades, a finding suggesting a significant lack of reliability of all three AI detection tools (Figure 1D).

In order to directly determine the false-positive and false-negative rates of three AI detection tools, a third experiment was designed. The experiment involved analyzing 28 abstracts written by the author, specifically chosen as true negatives since it could be reliably confirmed that no AI tools were used in their creation. To generate a data set that represented scientific texts modified by AI to enhance readability and language, these original abstracts were subsequently rewritten by ChatGPT. Additionally, a separate set of 28 entirely fabricated control abstracts was generated. These control abstracts, considered true positives, were created by providing ChatGPT with only the author's name and the abstract title. Manual screening verified that all the made-up abstracts were composed of text that sounded plausible but was factually incorrect. The abstracts were analyzed with three AI detection tools: the OpenAI classifier, GPTZero, and ZeroGPT detectors. Among these, the OpenAI classifier exhibited the most conservative behavior and the lowest sensitivity. While it correctly classified the original abstracts as human-written, it mistakenly identified AI-rewritten abstracts as human-written text. It only managed to correctly classify a single completely fabricated abstract (Figure 1E). GPTZero showed relatively better performance. It accurately classified the majority of original abstracts and successfully detected AI content in a large subset of both the rewritten and fabricated abstracts. However, a considerable number of AI-rewritten abstracts were still mislabeled as “likely entirely human” (Figure 1E). In contrast, ZeroGPT provided less conservative estimates, as was the case with the AD data set. It demonstrated the highest sensitivity as it correctly classified the majority of AI abstracts. However, it had the poorest specificity, as numerous original abstracts were mistakenly identified as AI texts (Figure 1F).

The results of this study were derived from a small proof-of-concept investigation, which was constrained by the limitations of the free application programming interface solutions. Therefore, these findings should be interpreted with caution. The generalizability of the data sets used in this study is possibly limited since AD abstracts may not accurately represent abstracts from other scientific fields. Additionally, the evaluation of the performance of AI detection tools may be subject to bias since the data set consisted solely of abstracts from a single author. Therefore, more extensive and rigorous studies should be performed to evaluate the conclusions drawn from this investigation.

In summary, the initial screening of recent AD abstracts using ZeroGPT indicated a widespread use of AI in writing scientific abstracts. However, the findings from the OpenAI classifier and GPTZero, and a temporal analysis with all three AI detection tools contradicted this notion, suggesting no substantial evidence of extensive AI usage in scientific abstract writing. The inconsistency of results obtained from different AI detection tools clearly highlights the inadequacy of current methods for accurately detecting AI-generated scientific abstracts. This raises the question of how publishers will identify ethical transgressions once the limits of AI use in scientific writing are defined.

## References

[1] Homolak J Opportunities and risks of ChatGPT in medicine, science, and academic publishing: a modern Promethean dilemma. Croat Med J 2023 64 1 3 10.3325/cmj.2023.64.1 36864812 PMC10028563

[2] Blanco-González AA Cabezón A Seco-González D Conde-Torres P Antelo-Riveiro Á Piñeiro A The role of AI in drug discovery: challenges, opportunities, and strategies. Pharmaceuticals (Basel) 2023 16 891 10.3390/ph16060891 37375838 PMC10302890

[3] van Dis EAM Bollen J Zuidema W van Rooij R Bockting CL ChatGPT: five priorities for research. Nature 2023 614 224 6 10.1038/d41586-023-00288-7 36737653

[4] Liebrenz MR Schleifer A Bhugra BD Smith A Generating scholarly content with ChatGPT: ethical challenges for medical publishing. Lancet Digit Health 2023 5 e105 6 10.1016/S2589-7500(23)00019-5 36754725

[5] Gao CA Howard FM Markov NS Dyer DS Ramesh S Luo Y Comparing scientific abstracts generated by ChatGPT to real abstracts with detectors and blinded human reviewers. NPJ Digit Med 2023 6 75 10.1038/s41746-023-00819-6 37100871 PMC10133283

